# Blood type A associates with critical COVID-19 and death in a Swedish cohort

**DOI:** 10.1186/s13054-020-03223-8

**Published:** 2020-08-12

**Authors:** Michael Hultström, Barbro Persson, Oskar Eriksson, Miklos Lipcsey, Robert Frithiof, Bo Nilsson

**Affiliations:** 1 Anesthesia and Intensive Care Medicine, Department of Surgical Sciences, Uppsala University, Uppsala, Sweden; 2 Integrative Physiology, Department of Medical Cell Biology, Uppsala University, Uppsala, Sweden; 3 Clinical Chemistry, Department of Immunology, Genetics and Pathology, Uppsala, Sweden

To the editor,

Coronavirus disease 2019 (COVID-19) is primarily associated with respiratory failure, but both renal and circulatory failure is common in patients that require critical care or die of the disease [1]. A recently published GWAS showed a strong association between severe COVID-19 and the ABO blood group locus in a cohort from Italy and Spain, with a higher risk for patients with blood group A [2]. This is consistent with data on susceptibility to COVID-19 being associated with blood group in Chinese [3]. Although the GWAS dataset did not include data on mortality, there is a preliminary report in an American cohort that blood group A is associated with both severity of COVID-19 and death [4].

Here, we present data on blood type distribution from a Swedish critical care cohort (*n* = 64) compared to the blood type distribution in the population as a whole: A 43%, AB 4%, B 9%, and O 44% giving a dichotomized distribution of A/AB 47% to B/O 53% [5]. Blood typing was performed using routine clinical procedures at the Clinical Immunology and Transfusion Medicine at the University Hospital in Uppsala.

Using the population blood type distribution to estimate the expected frequencies, we found an association of blood type A with the risk of requiring critical care (HR (95% CI) = 2.01 (1.23–3.28)) and increased risk of death within 30 days (HR (95% CI) = 3.16 (1.28–7.77)) in COVID-19 (Fig. 1). This indicates that the findings in the previous studies are consistent also in a northern European population.

**Fig. 1:**
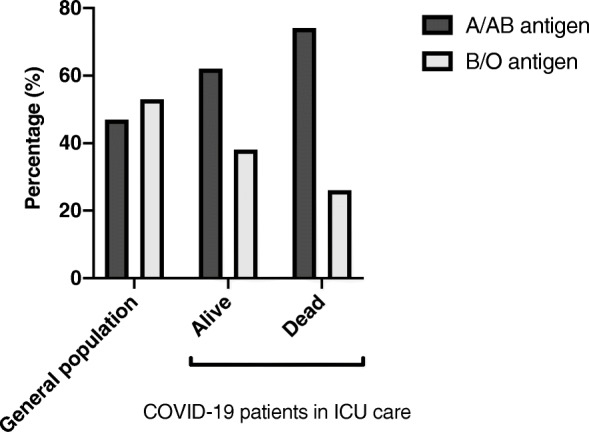
Distribution of blood types A/AB antigen and B/O antigen in the Swedish population (A/AB 47%, B/O 53%) compared to intensive care patients who survived (*n* = 45, A/AB 62%, B/O 38%) and who died (*n* = 19, A/AB 74%, B/O 26%) of COVID-19 at a tertiary care critical care facility in Sweden. Blood type A/AB is associated with an increased risk of death: hazard ratio (95% CI) = 3.16 (1.28–7.77)

Although the present study does not investigate the mechanism behind the association, one potential mechanism that may be involved in the increased risk linked to blood type antigen A is that it contains a galactose as end group saccharide. Both blood type B and O have a galactose amine in this position, which may explain the difference between the blood groups. The spike protein of SARS-COV-2 has been shown to bind carbohydrates, and a strong affinity between the A antigen and the virus could facilitate uptake of the virus into the cells [6].

Limitations of the present dataset is a relatively low number of patients, which in this case goes with a difference in the average age of 63 years for the A/AB group compared to 55 years for the B/O group. In addition, there is a difference in sex distribution of 81% male in the A/AB and 69% in the B/O. Both of these effects would tend to lead to an overestimation of the effect of blood type A/AB in the present dataset, which would tend to bring the results more in line with the previously published risk profile [6].

In conclusion, we show that blood type A or AB is associated with an increased risk of requiring critical care or dying of COVID-19 in the Swedish population. Taken together, with previously published data, this indicates that blood group A is a risk factor for disease severity and death in COVID-19 irrespective of the genetic background.

## Data Availability

Data in the current study is available from the corresponding author on a reasonable request.
